# Immigrant and ethnic neighbourhood concentration and reduced child developmental vulnerability: A Canadian cohort study

**DOI:** 10.23889/ijpds.v5i1.1147

**Published:** 2020-02-26

**Authors:** DN McRae, N Muhajarine, M Janus, E Duku, M Brownell, B Forer, M Guhn

**Affiliations:** 1 University of Saskatchewan; 2 McMaster University; 3 University of Manitoba; 4 University of British Columbia

## Abstract

**Introduction:**

Studies have consistently demonstrated a gradient between median neighbourhood income and child developmental outcomes. By investigating statistical outliers—neighbourhoods with children exhibiting less or more developmental vulnerability than that predicted by median neighbourhood income—there is an opportunity to identify other neighbourhood characteristics that may be enhancing or impeding early childhood development.

**Objective:**

Testing a variety of neighbourhood factors, including immigrant or ethnic concentration and characteristics of structural disadvantage (proportion of social assistance recipients, homes in need of major repair, residents with high school education only, lone parent families, and residents moving in the last year) we sought to identify factors associated with more or less developmental vulnerability than that predicted by median neighbourhood income, for young children.

**Methods:**

For this cross-sectional study we used validated Early Development Instrument (EDI) data (2003-2013) linked to demographic and socioeconomic Census and Tax Filer data for 98.3% of Canadian neighbourhoods (n=2,023). The purpose of the instrument is to report, at a population-level, children’s school readiness. Children’s developmental vulnerability was assessed in five domains (physical health and well-being, emotional maturity, social competence, language and cognitive development, and communication and general knowledge) in relation to the 10th percentile from a national normative sample. Levels of children’s neighbourhood vulnerability were determined per domain, as percent of children vulnerable at a given domain. Neighbourhoods were grouped into three cohorts, those having lower than predicted, as predicted, or higher than predicted children’s vulnerability according to neighbourhood median income. Using multivariable binary logistic regression we modelled the association between select neighbourhood characteristics and neighbourhoods with lower or higher than predicted vulnerability per domain, compared to neighbourhoods with predicted vulnerability. This allowed us to determine neighbourhood characteristics associated with better or worse child developmental outcomes, at a neighbourhood-level, than that predicted by income.

**Results:**

In neighbourhoods with less child developmental vulnerability than that predicted by income, high or low immigrant concentration and ethnic homogeneity was associated with less vulnerability in physical (adjusted odds ratio (aOR) 1.66, 95% CI: 1.43, 1.94), social (aOR 1.30, 95% CI: 1.11, 1.51), and communication domains (aOR 1.24, 95% CI: 1.03, 1.47) compared to neighbourhoods with vulnerability concordant with income. Neighbourhood ethnic homogeneity was consistently associated with less developmental vulnerability than predicted by income across all developmental domains. Neighbourhood-level structural disadvantage was strongly associated with child developmental vulnerability beyond that predicted by median neighbourhood income.

**Conclusion:**

Canadian neighbourhoods demonstrating less child developmental vulnerability than that predicted by income have greater ethnic and ethnic-immigrant homogeneity than neighbourhoods with child developmental vulnerability concordant with income. Neighbourhood social cohesion and cultural identity may be contributing factors. Neighbourhood structural disadvantage is associated with poorer early childhood development, over and above that predicted by neighbourhood income. Neighbourhood-level policy and programming should address income and non-income related barriers to healthy child development

## Findings

- Canadian neighbourhoods with less child developmental vulnerability than that predicted by income are characterized by ethnic and ethnic-immigrant homogeneity
- Within these neighbourhoods, social cohesion and support for cultural identity may be fostering child development
- Neighbourhood-level structural disadvantage is associated with poor developmental outcomes, independent of that predicted by median neighbourhood income
- Results suggests neighbourhood-level policy and programming should address both income and non-income related barriers to healthy child development

## Background

In Canada, a large proportion of variation in children’s developmental vulnerability (32%) has been associated with neighbourhood socioeconomic status (SES) [1]. There is a consistent gradient between neighbourhood income and developmental outcomes, with children living in low income neighbourhoods performing more poorly than their peers in physical, social, emotional, language, and communication domains [2, 3]. However, not all neighbourhoods have developmental outcomes congruent with their income level; in some neighbourhoods, developmental outcomes supersede income levels, and in other places they fall short of income-predicted expectations. Investigating these statistically outlying neighbourhoods—those with children exhibiting less or more developmental vulnerability than that predicted by median neighbourhood income—can provide clues as to neighbourhood characteristics other than income that enhance or diminish child development.

Evidence in the literature suggests that neighbourhood ethnic and immigrant concentration may be independently associated with healthy childhood development [4], mediated by social cohesion. A Canadian study of over 13,000 children aged four to 11 using parent and teacher assessments found lower levels of emotional-behavioural problems for children from recent immigrant families living in neighbourhoods of high immigrant concentration, compared to non-immigrant children, despite greater exposure to poverty [5]. In another study including over 5,000 children in rural and urban settings in British Columbia, Canada, children residing in neighbourhoods with high immigrant concentration demonstrated better language, numeracy, reading, and communication skills, depending on rurality and the child’s age at data collection [6]. In areas of immigrant concentration, neighbours’ mutual understanding of the challenges faced in learning a language, adopting new customs, coping with the loss of former social networks, and re-building new ones may deepen residents’ commitment to their local communities.

It has been postulated that neighbourhoods with high ethnic homogeneity have high levels of social interaction leading to ‘in-group bonding’ [7] and in turn greater social cohesion [8-10]. Social cohesion (as defined in this study) refers to the presence of strong social connections, trust, and mutual values between neighbours, and the absence of social tension (e.g., racism) [11, 12]. The benefits of neighbourhood social cohesion for child development have been demonstrated in a Canadian study drawing on a national sample of four- and five-year-old children (n=3,350), in which neighbourhood social cohesion (measured on a five-item scale) was associated with improved verbal ability and fewer child behavioural problems [4]. In addition, a recent review [13] of neighbourhood effects and early child development showed that poor relations among neighbours (indicated by social disorder, lack of belonging, low social cohesion, and diminished opportunity for community involvement) correlated with low language, emotional and behavioural child development in several studies [14-19].

A second area of interest for this study is assessing whether neighbourhood structural disadvantage contributes to children’s developmental vulnerability over and above that predicted by income. Structural disadvantage pertains to the systematically inequitable distribution of resources and opportunities among individuals and groups due to policies and governance, as well as social norms and values, defined by the dominant culture [20]. Social disorganization experts suggest neighbourhoods with high levels of structural disadvantage (operationalized as low SES, residential instability, lone parent families, etc.) have weaker social ties, resulting in fewer formal and informal social controls which help in upholding and reinforcing community norms and expectations [21] and provide emotional and psychological support for children and parents [13]. For instance, when neighbourhoods have high levels of dilapidated housing (because of weak regulation of tenants’ rights) or transient residents (due to high unemployment), fear and stress related to safety concerns and mistrust among neighbours can limit interaction and contribute to a negative social environment [22]. In these areas, neighbourhood adults may have little influence and authority among children due to a lack of familiarity [21]. Children growing up in neighbourhoods with structural disadvantage may have fewer educational and recreational opportunities due to the volume, quality, and safety of neighbourhood public facilities [23], cost of services and transportation, and fewer adults in their neighbourhood voluntarily engaging in local civic activities [24].

## Hypotheses

We hypothesized that neighbourhoods with less child developmental vulnerability than predicted by median income would be characterized by high immigrant concentration and low ethnic diversity. Secondly, we hypothesized that neighbourhoods with more child developmental vulnerability than predicted by median income would be characterized by structural disadvantage.

## Method

### Data Sources

This research is a part of the *Canadian Neighbourhoods and Early Child Development* (CanNECD) study [25]. Data were obtained from the Early Development Instrument (EDI) database housed at the Offord Centre for Child Studies, McMaster University [26]. Data are collected for individual children and then aggregated and analyzed at the neighbourhood-level. The purpose of the instrument is to report, at a population-level, children’s school readiness [27]. The instrument’s validity and reliability has been consistently demonstrated among different groups of children [28-31] including those with English-as-a-Second-Language [32].

The EDI is completed by classroom teachers in the second half of the student’s kindergarten year (in Canada, that is the year before entry to Grade 1, which children start attending the year they turn five) for each child in the class. The 103 EDI questions pertain to five developmental domains including physical health and well-being (e.g., “Is the child able to manipulate objects?”), social competence (e.g., “Is the child able to work independently?”), emotional maturity (e.g., “Will the child comfort a child who is crying or upset?”), language and cognitive development (e.g., “Can the child count to 20?”), and communication skills and general knowledge (e.g., “Does the child show interest in general knowledge about the world?”). See the full EDI questionnaire at https://edi.offordcentre.com/wp/wp-content/uploads/2019/01/EDI-ON-ENG-2018.pdf. Using the child’s individual score in each domain, vulnerability is determined (yes/no) in relation to the 10th percentile from a national normative sample. When data are aggregated based on children’s residence, neighbourhood children’s vulnerability is defined as the percent of children vulnerable in a specific domain per neighbourhood [27].

The EDI has been administered in 12 of the 13 Canadian provinces and territories over multiple waves [28] with de-identified, individual-level EDI scores aggregated to the neighbourhood-level for 2,058 neighbourhoods [25]. For this study we used the first wave of eligible data collected by each province/territory between 2003/2004 and 2012/2013. A data collection wave is equivalent to a provincial kindergarten census and can take up to three years to complete.

Boundaries of EDI neighbourhoods were drawn through consultation with government departments, community agencies and organizations, and academic groups to reflect locally defined borders and to potentially capture significant neighbourhood effects, as described in the CanNECD protocol [25]. Each neighbourhood was given a unique ID number. EDI scores were linked by postal code of a child’s residence to a CanNECD EDI neighbourhood and aggregated to the neighbourhood level. A data file with sociodemographic data from Canadian Census (2006 and 2011) and income tax (‘Tax Filer’) databases (2005 and 2010) aggregated to the same customized CanNECD EDI neighbourhoods was linked with the aggregated EDI data by neighbourhood ID [25].

For each province and territory, we selected Census and Tax Filer data from the year corresponding to the closest date of EDI data collection. In instances when EDI data collection fell mid-way between Censuses, the earlier Census/Tax Filer data were used, as they represented the sociodemographic conditions in which children were being raised prior to data collection. 

### Outcome Variables and Study Design

Using neighbourhood-level rates of child vulnerability in each of the five developmental domains we identified three types of neighbourhoods as our outcome variables:

1. those in which vulnerability rates were less than predicted by neighbourhood income (as defined below), having ‘discordant-lower vulnerability’;
2. those in which vulnerability rates were more than predicted by neighbourhood income, having ‘discordant-higher vulnerability’; and
3. those in which vulnerability rates were concordant with neighbourhood income, deemed ‘concordant vulnerability’.

Each of the two types of discordant neighbourhoods were compared to concordant neighbourhoods, coded as binary outcomes, to determine if the characteristics of interest were associated with less, or more, child developmental vulnerability at a neighbourhood-level than that predicted by income, indicative of a protective or a detrimental neighbourhood effect.

All five outcome domains were examined as previous studies have shown an association between at least one of the predictors examined (i.e., immigrant concentration) and children’s language, numeracy, reading, communication, emotional and behavioural outcomes [5, 6].

To classify neighbourhoods, we used methods introduced by Kershaw et al. [33]. We regressed the neighbourhood vulnerability rate (defined as percentage of children vulnerable at a specific domain) on neighbourhood median family income to produce residuals, or scores indicating the difference between the predicted vs. actual vulnerability rate. Kershaw et al. identified concordant and discordant neighbourhoods by selecting 50 neighbourhoods with the largest negative residuals and 50 neighbourhoods with the largest positive residuals among 478 British Columbia neighbourhoods, and compared these to 120 neighbourhoods with vulnerability scores closest to predicted scores. We modified this approach to capitalize on all available data and thus increase our sample size and power to detect differences in effect. We selected neighbourhoods with residuals in the lowest and highest quintiles (20% and 80% cut-off). Neighbourhoods with negative residuals, in the bottom 20th percent of all residual scores, were classified as having less developmental vulnerability than predicted by neighbourhood income (discordant-lower vulnerability). Neighbourhoods with the residual cut-off being greater than the 80th percentile were classified as more vulnerable than predicted (discordant-higher vulnerability). Discordant neighbourhoods were compared to concordant neighbourhoods, those with residuals between the 20th and 80th percentiles. 

### Neighbourhood-Level Predictors

Previous studies investigating neighbourhood structural disadvantage have included measures of neighbourhood income or concentrated poverty [34, 35], education [34], family structure (e.g., female-headed families) [34], and residential instability [21, 35]. Similarly, we operationalized structural disadvantage as the percent of neighbourhood residents receiving social assistance, dwellings in need of major repair, adults (25 to 64 years old) with high school completion or the equivalent as their highest credential, lone parent families, and residents having moved within the last year.

We created two indices based on previous methods for immigrant concentration [6] and ethnic diversity [36, 37]. For the immigrant concentration index, we used the mean of equally weighted z-scores for the Census variables, “percent with a non-official language as a mother tongue” and “percent that immigrated within the past 5 years”. Immigrants who reported arriving in Canada between 2001 and 2011 were included in the immigrant concentration index.

To measure ethnic diversity we used the fractionalization index, the simplest and most common index, which has been evaluated in contrast to more complex ethnicity indices and shown to have equivalent results [38]. The ethnic diversity index was created using a self-reported “ethnic origin” variable, available in the Census data. Ethnicities were classified as originating in seven geographic regions corresponding with Statistics Canada classifications: 1) North America, Britain, Western and Northern Europe; 2) South America, Central America, Caribbean; 3) Eastern, Southern, and Other Europe; 4) Africa; 5) West Central Asia, Middle East; 6) Eastern Asia, Southeast Asia, Southern Asia; and 7) Oceania [39].

To create ethnic diversity scores for all neighbourhoods, the percent of respondents indicating ethnicity from each region was calculated, squared and summed and the total subtracted from one [36]. Census respondents could indicate more than one ethnicity, therefore ethnic origins could total greater than 100% per neighbourhood. The ethnic diversity index had a range of values from 0 to 0.80 (M = 0.34; SD = 0.22). A neighbourhood ethnic diversity score of 0.34, for example, indicated a 34% likelihood that two randomly selected ethnic groups were different, when drawn from a pool of all weighted ethnic groups identified in a neighbourhood. Zero ethnic diversity indicated total ethnic homogeneity.

Because the predictors were measured on different scales, all values were z-score standardized—converted to standard deviations above or below the mean (which was set at 0)—to allow estimation of each variable’s relative contribution to the model. 

### Analysis Strategy

**Figure 1: Flow chart illustrating the number of neighbourhoods included in each domain-specific analysis:**
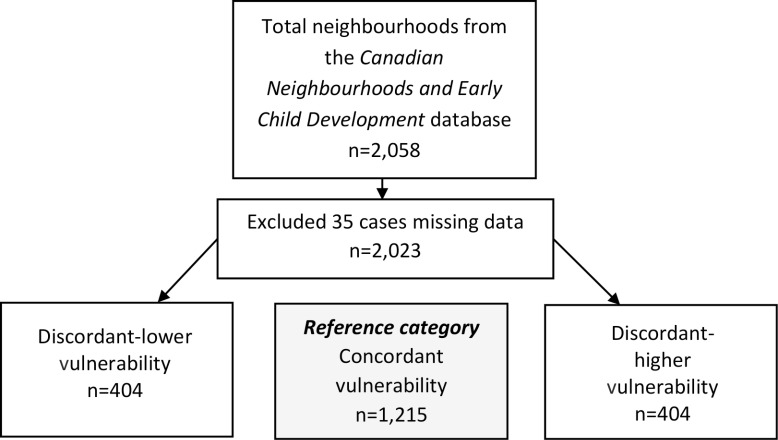


Our sample included 2,023 neighbourhoods with complete neighbourhood-level data. For each EDI domain we modelled the selected neighbourhood characteristics for 404 discordant-lower vulnerability neighbourhoods versus 1,215 concordant neighbourhoods, and 404 discordant-higher vulnerability neighbourhoods versus 1,215 concordant neighbourhoods (Figure 1).

Predictor variables were continuous (e.g., percent of neighbourhood dwellings in need of major repair) and outcome variables were dichotomous (e.g., discordant-lower vulnerability neighbourhood (Yes) versus concordant vulnerability neighbourhood (No)). To explore differences in mean neighbourhood characteristics for discordant-lower or discordant-higher vulnerability versus concordant vulnerability neighbourhoods we conducted independent t-tests. To test our hypotheses, we used multivariable binary logistic regression modelling, as the comparisons of interest were limited to neighbourhoods with discordant versus concordant vulnerability (rather than comparing discordant-lower with discordant-higher neighbourhoods). Only neighbourhoods with complete data (98.3%) were included in the analyses.

To select variables for the multivariable models, we conducted univariate binary logistic analyses, retaining variables of interest that had a Wald statistic p-value < 0.25. For our final variable selection, we used a manual backward elimination approach retaining variables with alpha values less than 0.05. Although our study included numerous statistical tests, we did not adjust the alpha level for multiple comparisons because the aim of our study was to explore if associations were consistent with the theoretical literature and showed promise for future mediation analyses. In this context, the risk of a Type I error—incorrectly rejecting the null hypothesis—due to multiple comparisons is of less concern than failing to identify potentially important associations worthy of more investigation [40].

Adjusted odds ratios and 95% confidence intervals are reported. Model fit was evaluated using the Hosmer-Lemeshow test (p > 0.05 indicates adequate fit) as well as estimates of outcome variance based on model predictors, assessed using Nagelkerke’s R2. SPSS Enterprise was used for data analysis [41].

Ethics approval for this study was granted by the University of British Columbia, Behavioural Research Ethics Board (registration number H13-00398). The research was of minimal risk as only aggregate neighbourhood-level data are reported. 

## Results

### Descriptive Results Neighbourhood characteristics and neighbourhood developmental vulnerability

Table 1 shows variation in mean rates of neighbourhood characteristics by neighbourhood vulnerability. For example, in neighbourhoods with discordant-lower developmental vulnerability in physical health and well-being (lower vulnerability than predicted by income) 7.15% of neighbourhood children were living in families receiving social assistance. A significantly greater proportion of children (7.95%) from concordant neighbourhoods (vulnerability similar to that predicted by income) were living in families receiving social assistance. Almost twelve per cent (11.69%) of children from discordant-higher vulnerability neighbourhoods (vulnerability higher than predicted by income) had families receiving social assistance, a statistically significant difference compared to rates among children from concordant neighbourhoods.

For three of the five domains (physical, social and communication), discordant-lower vulnerability neighbourhoods had significantly lower proportions of residents receiving social assistance, residents having high school as the highest credential, lone parents, and ethnic diversity, compared to concordant neighbourhoods. Unlike other domains, neighbourhoods that had discordant-lower vulnerability in emotional maturity did not have significantly different neighbourhood characteristics (aside from the proportion of lone parents) compared to concordant neighbourhoods.

Across all domains, discordant-higher vulnerability neighbourhoods had significantly higher mean rates of social assistance, dwellings in need of major repair, high school completion as the highest credential, lone parents, and residents that moved within the last year, compared to concordant neighbourhoods. Immigrant concentration was greater in neighbourhoods that had discordant-higher vulnerability in the language and cognitive development and the communication and general knowledge domains, compared to concordant vulnerability neighbourhoods.

**Table 1: Descriptive statistics for continuous variables by EDI domain and discordant-lower, discordant-higher, and concordant neighbourhood-level child developmental vulnerability, Canada (2003-2014):** Difference of means tested with independent t-tests; *p ≤ 0.05, ** p ≤ 0.01 **Definitions:** **social assistance:** percent of neighbourhood residents with low income receiving public financial aid; **dwelling major repair:** percent of neighbourhood residents living in a home requiring major repair; **high school education:** percent of neighbourhood residents 25 to 64 years old with high school completion or the equivalent as their highest credential; **lone parents:** percent of neighbourhood residents living in lone parent families; **moved in the last year:** percent of neighbourhood residents having moved in the last year; **immigrant index:** is derived from the standardized mean of two equally weighted variables, “percent of neighbourhood residents with a non-official language as a mother tongue” and “percent that immigrated within the past 5 years”. Values reported for immigrant index in this table are unstandardized for ease of interpretation.

		Neighbourhood Characteristics						
EDI domain	Neighbourhood vulnerability	Social assistance mean% (SD)	Dwelling major repair mean% (SD)	High school education mean% (SD)	Lone parents mean% (SD)	Moved in the last year mean% (SD)	Immigrant index mean% (SD)	Ethnic diversity index mean% (SD)
Physical health and well-being	Discordant-low	**7.15	2.86	**23.08	*14.56	**12.18	16.72	**0.29
	(n=404)	(4.38)	(1.60)	(4.50)	(4.74)	(5.43)	(13.18)	(0.25)
	Concordant	7.95	2.79	25.20	15.12	13.29	16.92	0.36
	(n=1,214)	(4.97)	(1.60)	(4.80)	(5.15)	(5.26)	(11.50)	(0.21)
	Discordant-high	**11.69	**3.81	**26.73	**17.71	**15.47	17.24	*0.33
	(n=404)	(6.87)	(1.97)	(4.07)	(6.33)	(5.54)	(11.36)	(0.18)
Social Competence	Discordant-low	**7.38	**3.11	**24.04	**14.46	**12.45	**14.92	**0.27
	(n=404)	(4.77)	(1.77)	(4.73)	(4.84)	(5.13)	(11.81)	(0.22)
	Concordant	8.21	2.84	25.04	15.34	13.40	17.27	0.36
	(n=1,214)	(5.19)	(1.64)	(4.80)	(5.27)	(5.46)	(11.49)	(0.22)
	Discordant-high	**10.67	**3.39	**26.23	**17.16	**14.86	17.99	0.35
	(n=404)	(6.59)	(1.88)	(4.33)	(6.14)	(5.49)	(12.60)	(0.21)
Emotional Maturity	Discordant-low	7.71	2.92	25.31	*14.59	13.31	17.84	0.34
	(n=404)	(5.13)	(1.80)	(4.80)	(5.42)	(5.73)	(12.93)	(0.23)
	Concordant	8.16	2.84	24.86	15.33	13.30	17.14	0.35
	(n=1,214)	(5.22)	(1.64)	(4.84)	(5.20)	(5.39)	(11.60)	(0.22)
	Discordant-high	**10.50	**3.58	*25.52	**17.06	**14.29	15.45	**0.29
	(n=404)	(6.34)	(1.78)	(4.36)	(5.85)	(5.32)	(11.20)	(0.20)
Language & cognitive development	Discordant-low	**7.26	2.99	**24.15	14.85	13.38	16.59	0.32
	(n=404)	(4.65)	(1.75)	(5.02)	(4.72)	(5.72)	(12.66)	(0.23)
	Concordant	8.18	2.84	25.06	15.16	13.11	16.39	0.34
	(n=1,214)	(5.20)	(1.58)	(4.65)	(5.22)	(5.26)	(11.28)	(0.22)
	Discordant-high	**10.87	**3.51	**26.06	**17.31	**14.80	**19.00	0.35
	(n=404)	(6.57)	(2.01)	(4.56)	(6.35)	(5.56)	(12.35)	(0.21)
Communication & general knowledge	Discordant-low	*7.40	3.04	**23.62	**14.46	**11.72	*11.53	**0.20
	(n=404)	(4.33)	(1.54)	(4.43)	(4.22)	(4.74)	(9.46)	(0.19)
	Concordant	8.06	2.91	25.28	15.21	13.31	16.34	0.35
	(n=1,214)	(5.15)	(1.67)	(4.81)	(5.30)	(5.42)	(10.86)	(0.21)
	Discordant-high	**11.12	**3.27	*25.92	**17.55	**15.88	**24.18	**0.44
	(n=404)	(6.80)	(2.03)	(4.55)	(6.38)	(5.41)	(13.18)	(0.20)

### Physical Health and Well-Being

In neighbourhoods with discordant-lower vulnerability in physical health (less vulnerability than predicted by income) there was less likelihood of residents receiving social assistance (aOR 0.73, 95%CI: 0.62, 0.85) and having only a high school education (aOR 0.70, 95% CI: 0.62, 0.80) compared to neighbourhoods with concordant or “as predicted by income” child developmental vulnerability (Table 2). These neighbourhoods were also characterized by high or low immigrant concentration and low ethnic diversity (aOR 1.66, 95% CI: 1.43, 1.94) compared to neighbourhoods with concordant vulnerability (Appendix 1: Figure 2a).

In neighbourhoods with discordant-higher physical vulnerability (more vulnerability than predicted by income), residents were more likely to have received social assistance (aOR 1.41, 95% CI: 1.23, 1.62), have dwellings in need of major repair (aOR 1.47, 95% CI: 1.27, 1.70), have only a high school education (aOR 1.44, 95% CI: 1.25, 1.65) and have moved in the last year (aOR 1.44, 95% CI: 1.26, 1.65) (Table 2) compared to neighbourhoods with developmental vulnerability concordant with neighbourhood income. In addition, there was a statistically significant interaction between immigrant concentration and ethnic diversity (aOR 0.82, 95% CI: 0.68, 0.98), with high immigrant concentration and low ethnic diversity best predicting the probability of a neighbourhood having discordant-higher vulnerability in physical health and well-being (Appendix 1: Figure 2b).

**Table 2: Results of multivariable logistic regression models of discordant-lower and discordant-higher neighbourhood-level developmental vulnerability (compared to concordant) in physical health and well-being regressed on neighbourhood-level predictors (Canada, 2003-2013):** Abbreviations: aOR adjusted odds ratio, CI confidence interval ^a^Discordant-lower vulnerability neighbourhoods had lower rates of child developmental vulnerability than predicted by neighbourhood median income, compared to concordant neighbourhoods. ^b^Discordant-higher vulnerability neighbourhoods had higher rates of child developmental vulnerability than predicted by neighbourhood median income, compared to concordant neighbourhoods. These analyses included 1,619 neighbourhoods; 404 discordant-lower vulnerability neighbourhoods versus 1,215 concordant vulnerability neighbourhoods, and 404 discordant-higher vulnerability neighbourhoods versus 1,215 concordant vulnerability neighbourhoods.

Neighbourhood-level predictors	Discordant-Lower Vulnerability Neighbourhoods^a^	Discordant-Higher Vulnerability Neighbourhoods^b^
	aOR (95% CI)	aOR (95% CI)

Social assistance	0.73 (0.62, 0.85)	1.41 (1.23, 1.62)
Dwellings in need of major repair	-----	1.47 (1.27, 1.70)
High school education as highest credential	0.70 (0.62, 0.80)	1.44 (1.25, 1.65)
Lone parent families	-----	-----
Moved in the last year	-----	1.44 (1.26, 1.65)
Immigrant concentration	1.72 (1.38, 2.14)	1.14 (0.91, 1.42)
Ethnic diversity	0.46 (0.38, 0.54)	0.88 (0.72, 1.08)
Immigrant concentration X Ethnic diversity	1.66 (1.43, 1.94)	0.82 (0.68, 0.98)
Hosmer-Lemeshow	0.24	0.47
Nagelkerke R2	0.15	0.19

### Social Competence

Neighbourhoods with discordant-lower vulnerability in social competence were 30% less likely to have residents receiving social assistance (aOR 0.70, 95% CI: 0.60, 0.83) and 19% more likely to have dwellings in need of major repair (aOR 1.19, 95% CI: 1.04, 1.37) compared to neighbourhoods in which rates of vulnerability were concordant with income (Table 3). These neighbourhoods also had significantly different immigrant and ethnic composition compared to concordant neighbourhoods (aOR 1.30, 95% CI: 1.11, 1.51) (Table 3). The combination of high immigrant concentration and low ethnic diversity best predicted the probability of a neighbourhood having discordant-lower vulnerability in social competence, followed by neighbourhoods with low immigrant concentration and low ethnic diversity (Appendix 1: Figure 3).

Neighbourhoods with discordant-higher vulnerability in social competence were more likely to have residents receiving social assistance (aOR 1.27, 95% CI: 1.12, 1.45), dwellings in need of major repair (aOR 1.21, 95% CI 1.06, 1.39), adults with high school education only (aOR 1.28, 95% CI: 1.13, 1.44), and residents having moved in the last year (aOR 1.22, 95% CI: 1.09, 1.37) compared to neighbourhoods in which developmental vulnerability was concordant with income (Table 3). There was no statistically significant effect associated with immigrant concentration or ethnic diversity when comparing these two types of neighbourhoods.

**Table 3: Results of multivariable logistic regression models of discordant-lower and discordant-higher neighbourhood-level developmental vulnerability (compared to concordant) in social competence regressed on neighbourhood-level predictors (Canada, 2003-2013):** Abbreviations: aOR adjusted odds ratio, CI confidence interval ^a^Discordant-lower vulnerability neighbourhoods had lower rates of child developmental vulnerability than predicted by neighbourhood median income, compared to concordant neighbourhoods. ^b^Discordant-higher vulnerability neighbourhoods had higher rates of child developmental vulnerability than predicted by neighbourhood median income, compared to concordant neighbourhoods. These analyses included 1,619 neighbourhoods; 404 discordant-lower vulnerability neighbourhoods versus 1,215 concordant vulnerability neighbourhoods, and 404 discordant-higher vulnerability neighbourhoods versus 1,215 concordant vulnerability neighbourhoods.

Neighbourhood-level predictors	Discordant-Lower Vulnerability Neighbourhoodsa	Discordant-Higher Vulnerability Neighbourhoodsb
	aOR (95% CI)	aOR (95% CI)
Social assistance	0.70 (0.60, 0.83)	1.27 (1.12, 1.45)
Dwellings in need of major repair	1.19 (1.04, 1.37)	1.21 (1.06, 1.39)
High school education as highest credential	-----	1.28 (1.13, 1.44)
Lone parent families	-----	-----
Moved in the last year	-----	1.22 (1.09, 1.37)
Immigrant concentration	1.41 (1.15, 1.73)	-----
Ethnic diversity	0.54 (0.46, 0.65)	-----
Immigrant concentration X Ethnic diversity	1.30 (1.11, 1.51)	-----
Hosmer-Lemeshow	0.70	0.54
Nagelkerke R2	0.08	0.08

### Emotional Maturity

Neighbourhoods with discordant-lower vulnerability in emotional maturity were 17% more likely to have residents with a high school diploma as their highest credential (aOR 1.17, 95% CI: 1.04, 1.31) and 19% less likely to have families headed by lone parents (aOR 0.81, 95% CI: 0.71, 0.93) compared to neighbourhoods with rates of emotional vulnerability concordant with income (Table 4). These neighbourhoods were also characterized by high or low immigrant concentration and low ethnic diversity, or high immigrant concentration and high ethnic diversity (aOR 1.19, 95% CI: 1.02, 1.38) (Appendix 1: Figure 4).

Neighbourhoods with discordant-higher vulnerability in emotional maturity were 33% more likely to have residents who were receiving social assistance (aOR 1.33, 95% CI: 1.17, 1.53) (Table 4), 19% more likely to have dwellings in need of major repair (aOR 1.19, 95% CI: 1.03, 1.37), and 27% more likely to have residents that had moved within the last year (aOR 1.27, 95% CI: 1.12, 1.45) compared to neighbourhoods in which children’s emotional vulnerability was concordant with income. Ethnic diversity was also less likely in these neighbourhoods compared to concordant neighbourhoods (aOR 0.68, 95% CI: 0.59, 0.79).

**Table 4: Results of multivariable logistic regression models of discordant-lower and discordant-higher neighbourhood-level developmental vulnerability (compared to concordant) in emotional maturity regressed on neighbourhood-level predictors (Canada, 2003-2013):** Abbreviations: aOR adjusted odds ratio, CI confidence interval ^a^Discordant-lower vulnerability neighbourhoods had lower rates of child developmental vulnerability than predicted by neighbourhood median income, compared to concordant neighbourhoods. ^b^Discordant-higher vulnerability neighbourhoods had higher rates of child developmental vulnerability than predicted by neighbourhood median income, compared to concordant neighbourhoods. These analyses included 1,619 neighbourhoods; 404 discordant-lower vulnerability neighbourhoods versus 1,215 concordant vulnerability neighbourhoods, and 404 discordant-higher vulnerability neighbourhoods versus 1,215 concordant vulnerability neighbourhoods.

Neighbourhood-level predictors	Discordant-Lower Vulnerability Neighbourhoodsa	Discordant-Higher Vulnerability Neighbourhoodsb
	aOR (95% CI)	aOR (95% CI)
Social assistance	-----	1.33 (1.17, 1.53)
Dwellings in need of major repair	-----	1.19 (1.03, 1.37)
High school education as highest credential	1.17 (1.04, 1.31)	-----
Lone parent families	0.81 (0.71, 0.93)	-----
Moved in the last year	-----	1.27 (1.12, 1.45)
Immigrant concentration	1.31 (1.06, 1.62)	-----
Ethnic diversity	0.83 (0.70, 0.98)	0.68 (0.59, 0.79)
Immigrant concentration X Ethnic diversity	1.19 (1.02, 1.38)	-----
Hosmer-Lemeshow	0.78	0.63
Nagelkerke R2	0.02	0.09

### Language and Cognitive Development

Neighbourhoods in which children had discordant-lower vulnerability in language and cognitive development than that predicted by income were less likely to have residents receiving social assistance (aOR 0.75, 95% CI: 0.65, 0.86) compared to concordant neighbourhoods (Table 5). When modelling immigrant concentration and ethnic diversity, there was a statistically significantly interaction (aOR 1.36, 95% CI: 1.17, 1.58) with discordant lower-vulnerability neighbourhoods having a greater probability of low ethnic diversity and low or high immigrant concentration, or high immigrant concentration and high ethnic diversity, compared to concordant neighbourhoods (Appendix 1: Figure 5).

Neighbourhoods with discordant-higher vulnerability in language and cognitive development were more likely to have social assistance recipients (aOR 1.20, 95% CI: 1.05, 1.37), dwellings in need of major repair (aOR 1.37, 95% CI: 1.20, 1.57), residents with high school education only (aOR 1.28, 95% CI: 1.12, 1.45), individuals having moved within the last year (aOR 1.19, 95% CI: 1.05, 1.36) and greater immigrant concentration (aOR 1.30, 95% CI: 1.10, 1.53) than neighbourhoods with vulnerability concordant with income (Table 5).

**Table 5: Results of multivariable logistic regression models of discordant-lower and discordant-higher neighbourhood-level developmental vulnerability (compared to concordant) in language and cognitive development regressed on neighbourhood-level predictors (Canada, 2003-2013):** Abbreviations: aOR adjusted odds ratio, CI confidence interval ^a^Discordant-lower vulnerability neighbourhoods had lower rates of child developmental vulnerability than predicted by neighbourhood median income, compared to concordant neighbourhoods. ^b^Discordant-higher vulnerability neighbourhoods had higher rates of child developmental vulnerability than predicted by neighbourhood median income, compared to concordant neighbourhoods. *For this model, “dwellings in need of major repair” was statistically significant (Wald p < 0.05) but we chose to exclude it because model fit was inadequate (Hosmer-Lemeshow p=0.01) with its inclusion. These analyses included 1,619 neighbourhoods; 404 discordant-lower vulnerability neighbourhoods versus 1,215 concordant vulnerability neighbourhoods, and 404 discordant-higher vulnerability neighbourhoods versus 1,215 concordant vulnerability neighbourhoods.

Neighbourhood-level predictors	Discordant-Lower Vulnerability Neighbourhoodsa*	Discordant-Higher Vulnerability Neighbourhoodsb
	aOR (95% CI)	aOR (95% CI)
Social assistance	0.75 (0.65, 0.86)	1.20 (1.05, 1.37)
Dwellings in need of major repair	-----	1.37 (1.20, 1.57)
High school education as highest credential	-----	1.28 (1.12, 1.45)
Lone parent families	-----	-----
Moved in the last year	-----	1.19 (1.05,1.36)
Immigrant concentration	1.25 (1.01, 1.53)	1.30 (1.10, 1.53)
Ethnic diversity	0.76 (0.64, 0.89)	-----
Immigrant concentration X Ethnic diversity	1.36 (1.17, 1.58)	-----
Hosmer-Lemeshow	0.15	0.98
Nagelkerke R2	0.03	0.10

### Communication Skills and General Knowledge

Neighbourhoods with discordant-lower vulnerability in communication skills and general knowledge were less likely to have residents with dwellings in need of major repair (aOR 0.82, 95% CI: 0.71, 0.95), and high school as their highest credential (aOR 0.67, 95% CI: 0.59, 0.76) (Table 6). In combination, immigrant concentration and ethnic diversity were statistically significantly associated with neighbourhood discordant-lower vulnerability in communication and general knowledge, compared to concordant neighbourhoods (aOR 1.24, 95% CI: 1.03, 1.47) (Table 6). Low or high immigrant concentration coupled with low ethnic diversity increased the probability of neighbourhoods having discordant-lower vulnerability compared to concordant neighbourhoods (Appendix 1: Figure 6).

Neighbourhoods with discordant-higher vulnerability in communication skills and general knowledge had higher odds of residents receiving social assistance (aOR 1.39, 95% CI: 1.24, 1.55), having high school completion as the highest credential (aOR 1.29, 95% CI: 1.13, 1.47), and having moved within the last year (aOR 1.27, 95% CI: 1.12, 1.45) compared to neighbourhoods with vulnerability concordant with income (Table 6). As well, these neighbourhoods were characterized by higher odds of immigrant concentration (aOR 2.01, 95% CI: 1.71, 2.37) compared to concordant neighbourhoods.

**Table 6: Results of multivariable logistic regression models of discordant-lower and discordant-higher neighbourhood-level developmental vulnerability (compared to concordant) in communication skills and general knowledge (Canada, 2003-2013):** Abbreviations: aOR adjusted odds ratio, CI confidence interval ^a^Discordant-lower vulnerability neighbourhoods had lower rates of child developmental vulnerability than predicted by neighbourhood median income, compared to concordant neighbourhoods. ^b^Discordant-higher vulnerability neighbourhoods had higher rates of child developmental vulnerability than predicted by neighbourhood median income, compared to concordant neighbourhoods. These analyses included 1,619 neighbourhoods; 404 discordant-lower vulnerability neighbourhoods versus 1,215 concordant vulnerability neighbourhoods, and 404 discordant-higher vulnerability neighbourhoods versus 1,215 concordant vulnerability neighbourhoods.

Neighbourhood-level predictors	Discordant-Lower Vulnerability Neighbourhoods^a^	Discordant-Higher Vulnerability Neighbourhoods^b^
	aOR (95% CI)	aOR (95% CI)
Social assistance	-----	1.39 (1.24, 1.55)
Dwellings in need of major repair	0.82 (0.71, 0.95)	-----
High school education as highest credential	0.67 (0.59, 0.76)	1.29 (1.13, 1.47)
Lone parent families	-----	-----
Moved in the last year	-----	1.27 (1.12,1.45)
Immigrant concentration	1.09 (0.86, 1.38)	2.01 (1.71, 2.37)
Ethnic diversity	0.38 (0.32, 0.46)	-----
Immigrant concentration X Ethnic diversity	1.24 (1.03, 1.47)	-----
Hosmer-Lemeshow	0.01	0.62
Nagelkerke R2	0.19	0.17

### Patterns of predictors across EDI domains

Immigrant concentration and ethnic diversity statistically significantly interacted across all domains in discordant-lower vulnerability neighbourhoods. Neighbourhoods with less vulnerability in physical, social, and communication skills and general knowledge were more likely to have high or low immigrant concentration and low ethnic diversity, compared to neighbourhoods in which children’s vulnerability was concordant with income. Neighbourhoods with discordant-lower vulnerability in emotional maturity, or language and cognitive development, were most often characterized by high or low immigrant concentration and low ethnic diversity, or high immigrant concentration and high ethnic diversity compared to concordant neighbourhoods. Across all five domains, low ethnic diversity emerged as the most consistent neighbourhood characteristic associated with less developmental vulnerability; levels of immigrant concentration only marginally influenced probabilities.

Interestingly, in discordant-higher vulnerability neighbourhoods, immigrant concentration was significantly associated with greater vulnerability in language and cognitive development (aOR 1.30, 95% CI: 1.10, 1.53) and communication skills and general knowledge (aOR 2.01, 95% CI: 1.71, 2.37) compared to neighbourhoods with vulnerability concordant with income. In discordant-higher vulnerability neighbourhoods the interaction between immigrant concentration and ethnic diversity was only statistically significant in the physical health and well-being domain (aOR 0.82, 95% CI: 0.68, 0.98); neighbourhoods with high immigrant concentration and low ethnic diversity were more likely to have discordant-higher vulnerability in physical health and well-being than concordant neighbourhoods.

For all EDI domains, neighbourhoods with discordant-higher developmental vulnerability had greater odds of individuals receiving social assistance and residents having moved in the last year. In addition, for all domains except communication skills and general knowledge, neighbourhoods with discordant-higher vulnerability were more likely to have dwellings in need of major repair. Across all EDI domains, with the exception of emotional maturity, neighbourhoods with discordant-higher vulnerability had greater odds of residents having high school graduation as their highest credential. Lone parent status, however, was not a statistically significant characteristic of discordant-higher vulnerability neighbourhoods, for any domain. 

### Model fit

Of the 10 models constructed, one had significantly poor fit (see Table 6). Nonetheless we have included results from all models because the study aim was to test the specified predictors against all EDI domains.

## Discussion

Neighbourhood immigrant concentration and ethnic composition was significantly associated with neighbourhood-level discordant-lower developmental vulnerability, compared to concordant vulnerability, for each of the five developmental domains. Neighbourhoods with discordant-lower vulnerability in physical health and well-being, social competence, and communication skills and general knowledge were characterized by either high or low levels of immigrant concentration and ethnic homogeneity. Across all five domains the most consistent neighbourhood characteristic associated with discordant-lower vulnerability was ethnic homogeneity, with levels of immigrant concentration only marginally influencing predicted probabilities. In other words, areas where inhabitants were of similar ethnicity had, on average, lower levels of child developmental vulnerability than those with diverse ethnicity. In two domains (language and cognitive development and communication skills and general knowledge), immigrant concentration predicted discordant-higher vulnerability. As hypothesized, neighbourhood-level structural disadvantage measured by low education, social assistance receipt, residential instability and residential decay was consistently associated with child developmental vulnerability beyond that predicted by median neighbourhood income.

This pan-Canadian, population-level study utilized robust area-level sociodemographic data, a validated tool for measuring child development, and a previously tested, innovative methodology [33] to investigate correlates of discordance and concordance of area-level childhood developmental vulnerability with income-based predictions. Few area-level studies account for both the influence of immigrant concentration and ethnic diversity in assessing area-level attributes contributing to health and well-being [42], despite the fact that new immigrants often prefer to settle in communities with others of the same ethnic origin [43]. This study adds to the literature by demonstrating both their independent and combined association with child development. Furthermore, we were able to verify meaningful associations between a number of neighbourhood-level risk factors and child developmental well-being that have previously been identified in the literature [16, 44-46].

### Ethnic homogeneity and immigrant concentration associated with developmental vulnerability

Our results indicated a pattern in the relationship between less developmental vulnerability than predicted by neighbourhood median income and ethnic homogeneity across three domains (physical, social, and communication). These results coincide with findings from the Netherlands and the UK [47, 48] where researchers similarly identified neighbourhoods with unexpectedly positive health outcomes despite socioeconomic risk—described as resilient—as having either a low proportion of non-western immigrants (European ethnic homogeneity) [47] or ethnic or religious homogeneity [48]. A Canadian study examining children’s developmental outcomes has shown a similar effect for immigrant children living in immigrant neighbourhoods [5].

One interpretation of our findings is that ethnic and ethnic-immigrant homogenous communities possess assets, aside from financial resources, that strengthen children’s ability to succeed at an early age. Socially cohesive ethnic neighbourhoods may provide unique opportunities for the transmission of advice on how to “get ahead” as a minority individual, indirectly impacting child development through access to community programs and resources [49]. In contrast to neighbourhood social networks built around common SES (e.g. middle-class families, living in middle-class neighbourhoods), neighbourhood networks based on ethnic origin may have greater socioeconomic heterogeneity because they are built around common culture, language, and heritage. Ethnic networks may, therefore, be more inclusive of lower SES families and individuals enabling them greater access to social, educational, and economic opportunities.

Furthermore, if ethnic neighbourhoods have strong social cohesion this could encourage neighbours to pool resources (e.g. housing, transportation, skills, and knowledge), providing day-to-day social and practical support as well as economic safety nets for emergency situations, thereby reducing stress [49]. First-generation Canadian immigrants living in areas of high immigrant concentration have been shown to have significantly less mood or anxiety disorders, substance-dependence and lifetime prevalence of psychotic disorder, compared to those living in less immigrant-dense areas [50]. Strong social and cultural networks among adult neighbours may promote emotional and psychological health, via social, emotional, financial, and practical support, enabling parents to invest in their child(ren)’s health and development.

Cultural identity may also be an important asset in neighbourhoods with high concentrations of immigrant residents or those of the same ethnicity. In a Canadian study of young immigrant and Canadian-born Chinese children, participation in Chinese cultural activities was significantly associated with increased capabilities in a variety of areas, including peer acceptance and teacher-rated competence, as well as decreased shyness and loneliness, and improved perceptions of self-worth [51]. Participation in Western cultural activities also increased teacher-rated competence, but participation in Chinese cultural activities appeared to have much further-reaching effects for social and psychological well-being. Likewise, in a 13 country study of adolescents 13 to 18 years of age, immigrant youth who indicated a clear sense of ethnic identity were better able to psychologically adapt to their host country, compared to their peers with low ethnic identity [52]. Adolescents were more likely to report an ethnic identity if they lived in neighbourhoods with high concentrations of residents of the same ethnic origin.

Cultural identity may also encourage children to conform to cultural values, beliefs, and expectations [10]. This may impact academic achievement as immigrant parents report placing high value on children’s academic success to compensate for career and social disadvantages associated with their minority status [53, 54]. In addition, immigrant children may be driven to succeed by cultural obligation and feelings of indebtedness, particularly when parents chose to migrate to provide greater educational and career opportunities for their children [55, 56]. When these concepts germinate from within a neighbourhood which has high social cohesion, such aspirations may present as a collective norm, helping to reinforce and promote communal values [57].

Country of origin has been shown to shape ethnic or ethnic-immigrant norms and expectations [53, 58] as well as educational achievement [10], therefore future research should examine the effects of neighbourhood ethnic homogeneity by ethnic group. In our study, Caucasian majority neighbourhoods would have been classified as having low ethnic diversity, yet if they displayed significantly less or more vulnerability than predicted there could be different underlying mechanisms driving the association than what we would expect for immigrant or minority groups. It is also important to note that our results may be due in part to Canadian immigration policy that favors healthy and financially secure immigrants [59]. Immigrant-ethnic communities, even those in low SES neighbourhoods, may be advantageous for child development due to Canadian immigrant selection policies, compared to neighbourhoods with lower immigrant concentration [60].

Neighbourhoods where there was discordant-higher vulnerability in language and cognitive development and communication skills and general knowledge were characterized by high immigrant concentration. These findings confirm what has been observed elsewhere; some studies have shown young immigrant children to have weaker language and communication skills compared to their non-immigrant peers [31, 61]. Depending on country of origin, children’s age at migration, and parents’ fluency in an official language, living in a neighbourhood with high immigrant concentration may reduce children’s contact with adults fluent in an official language. Additionally, children of the same linguistic background living in close proximity may choose to communicate among themselves using their native language, impacting language acquisition for English language learners. Our results emphasize the need for further study, using longitudinal designs, to assess how neighbourhood immigrant concentration impacts communication skills over time. It could be that neighbourhoods with high immigrant concentration have children with more vulnerability in the communication domain than predicted by income due to bilingual language development, but over time these children may catch up with their peers as suggested in other studies [62, 63].

Our results revealed two surprising findings, which did not align with our first hypothesis. Low ethnic diversity was associated with discordant-higher neighbourhood-level vulnerability in emotional maturity, and high immigration concentration coupled with low ethnic diversity predicted discordant-higher vulnerability in physical health and well-being. These results suggest neighbourhood immigrant-ethnic or ethnic concentration could exert both positive and negative effects on specific domains of child development at the neighbourhood-level. Alternatively, immigrant and/or ethnic concentration may interact with other individual or neighbourhood characteristics unmeasured in this study. Further study is warranted to explore potential explanations.

### Structural disadvantage as a predictor of developmental vulnerability

Our findings indicate that neighbourhood structural disadvantage is strongly associated with child developmental vulnerability over and above that predicted by income. Previous mediation analysis has demonstrated how multiple neighbourhood disadvantages can be indirectly associated with child behavioural problems via low neighbourhood social cohesion and an increase in prevalence of maternal depression and punitive parenting practices [34]. Likewise, neighbourhood residential instability in areas of structural disadvantage has been associated with poorer home learning environments, mediated by mothers’ perception of neighbourhood disorder and depressive symptoms [64]. These examples highlight how neighbourhood social organization in areas of structural disadvantage may be shaping adults’ and children’s behaviour, in turn impacting children’s developmental outcomes [65]. More research is needed to unpack, in varying contexts, the mediating factors to ensure that policy and program efforts addressing neighbourhood risk target outward measures of risk (e.g. residential decay) in a manner that mitigates the mediating factors (e.g. parents’ psychosocial health).

## Limitations

This study adds to the literature by demonstrating how specific, conceptually chosen neighbourhood factors contribute to young children’s development. However, it is important to note that the significant neighbourhood-level predictors identified only accounted for a portion of the variation evident in child developmental outcomes (Nagelkerke R2 ranged from 2% to 26% depending on the EDI domain). These results demonstrate that there are important neighbourhood-level factors shaping child development that should be considered in future studies, together with individual, household, and school characteristics as well as municipal, provincial and federal policy and environmental factors.

While using Canadian Census data allowed us to conduct this study at the population level, it also prevented us from fully understanding how individuals reported ethnic origin (e.g., the meaning respondents attached to the term). Presumably, ethnic origin was synonymous with ethnic identity for some individuals whereas it could have been a description of historical ancestry for others. This limits the interpretation of ethnic diversity/homogeneity and highlights the need for qualitative follow-up studies to explore the meaning of ‘ethnic origin’ and ethnic identity amongst Census respondents. 

## Conclusion

High or low immigrant concentration and ethnic homogeneity was associated with less likelihood of child developmental vulnerability at a neighbourhood-level. Ethnic or immigrant-ethnic neighbourhoods may be substantially different from other neighbourhoods. It may be that the benefits of shared language and culture (e.g. parenting styles and religious beliefs) encourage a sense of neighbourhood social cohesion, fostering children’s cultural identity, promoting child development. Future research is needed to specifically test social cohesion as a mechanism linking neighbourhood-level immigrant concentration and low ethnic diversity to less child developmental vulnerability. Research should also focus on which immigrant/ethnic groups are experiencing more favourable outcomes than predicted and under what conditions. Neighbourhood structural disadvantage, over and above income, appears to significantly impair achievement of child developmental milestones at an appropriate age. This suggests neighbourhood-level policy and programming should address both income and non-income related barriers to healthy child development. Our findings reassert the importance of both the social and physical environment in shaping early childhood development.

## Included Appendix

Supplementary Appendix 1: Figures illustrating the interaction between immigrant concentration and ethnic diversity for neighbourhoods with discordant-higher or -lower vulnerability, by domain

## Ethics statement

Ethics approval for this study has been granted by the University of British Columbia Behavioural Research Ethics Board (ID# H13-00398). Participant consent was not required as all child-level data was de-identified and findings are reported at an aggregated, neighbourhood level.

## Figures and Tables

**Figure 2a: Interaction between immigrant concentration and ethnic diversity for neighbourhoods with discordant-lower vulnerability in physical health and well-being versus concordant neighbourhoods (n=1,619)1:**
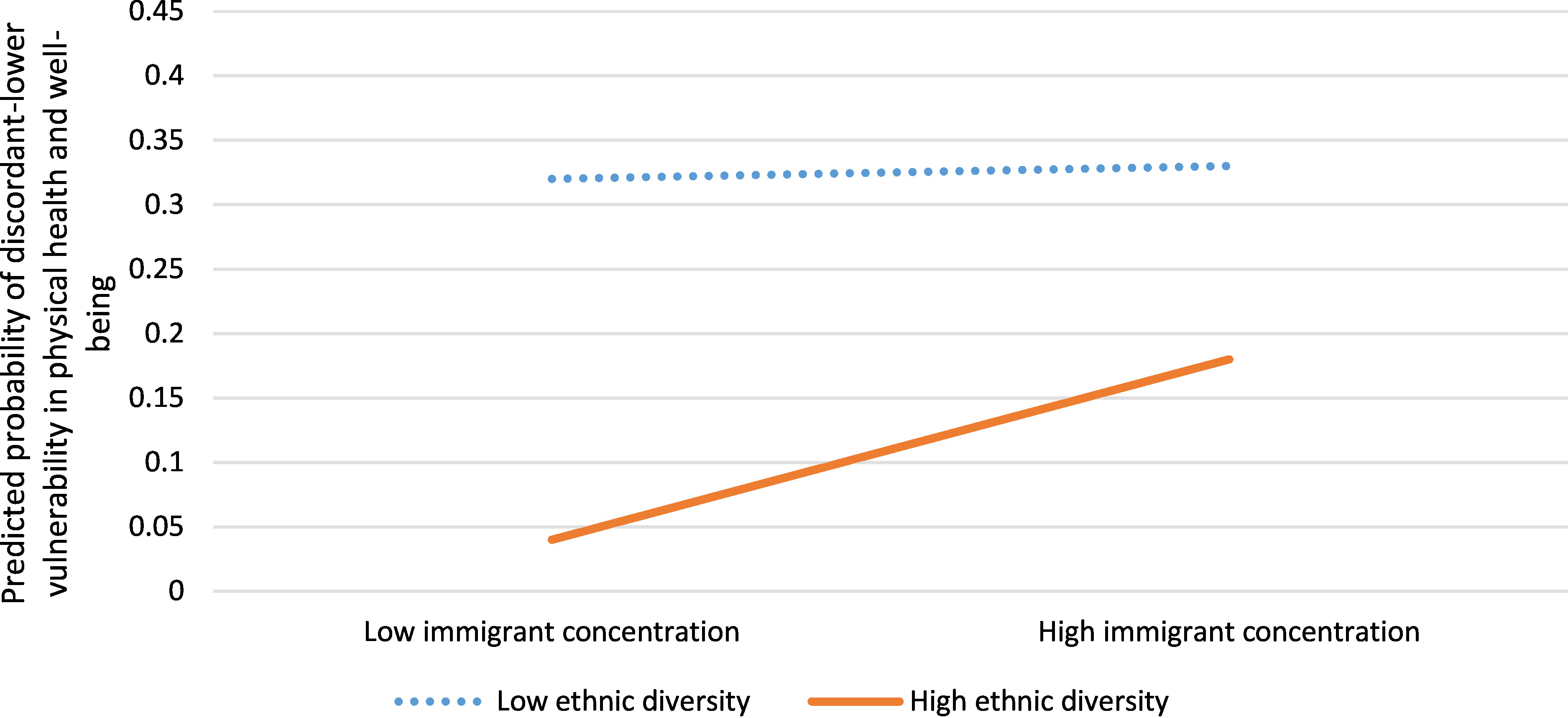
^1^ Figure legend for this and all proceeding figures: High/low ethnic diversity and high/low immigrant concentration are equal to one standard deviation above/below the mean for the respective variables

**Figure 2b: Interaction between immigrant concentration and ethnic diversity for neighbourhoods with discordant-higher vulnerability in physical health and well-being versus concordant neighbourhoods (n=1,619):**
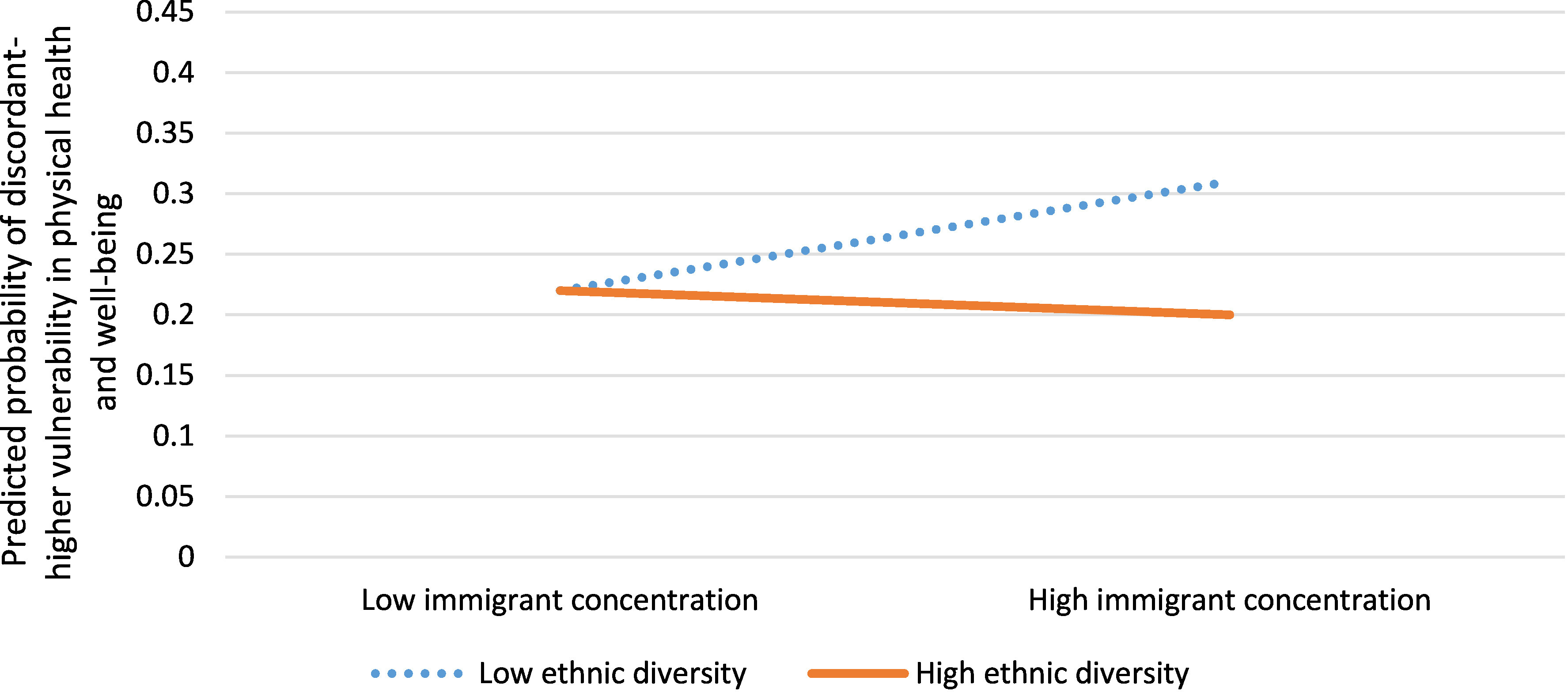


**Figure 3: Interaction between immigrant concentration and ethnic diversity for neighbourhoods with discordant-lower vulnerability in social competence versus concordant neighbourhoods (n=1,619):**
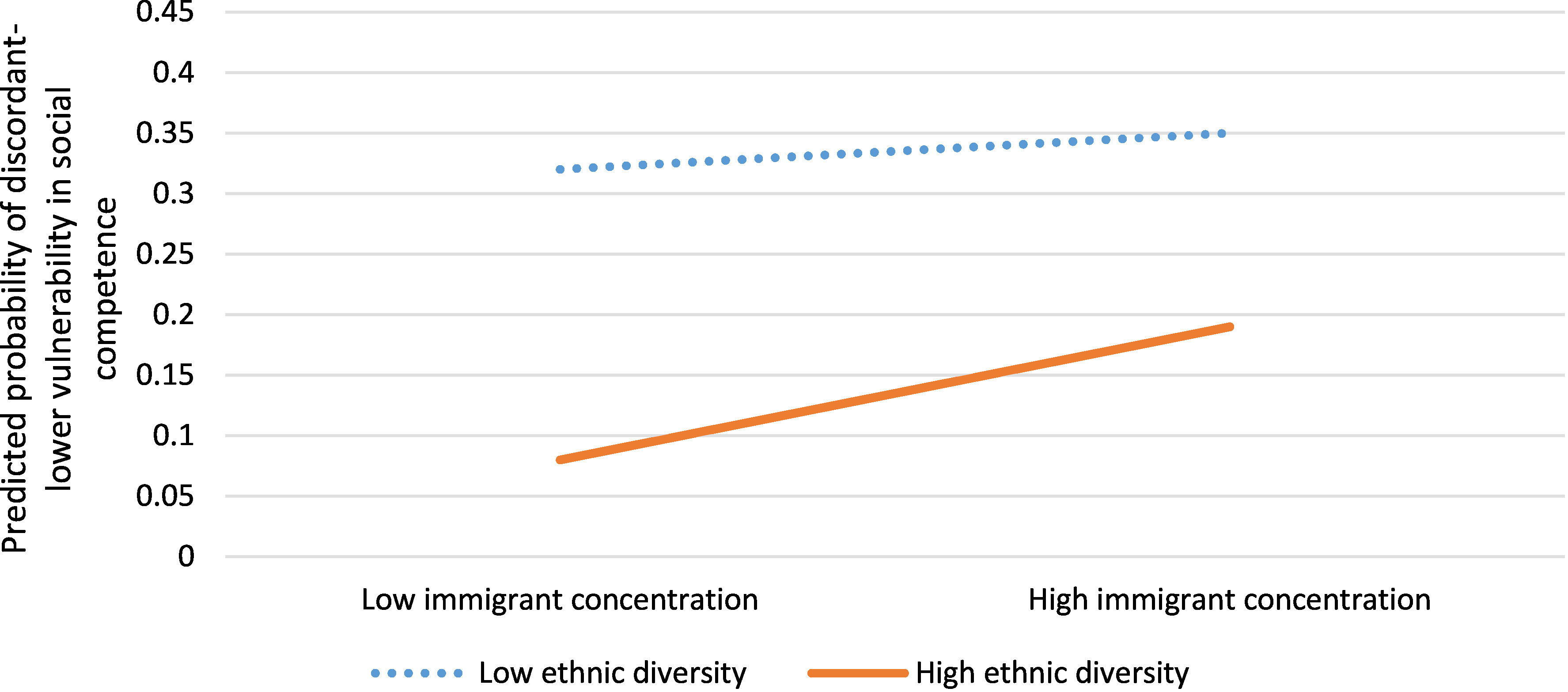


**Figure 4: Interaction between immigrant concentration and ethnic diversity for neighbourhoods with discordant-lower vulnerability in emotional maturity versus concordant neighbourhoods (n=1,619):**
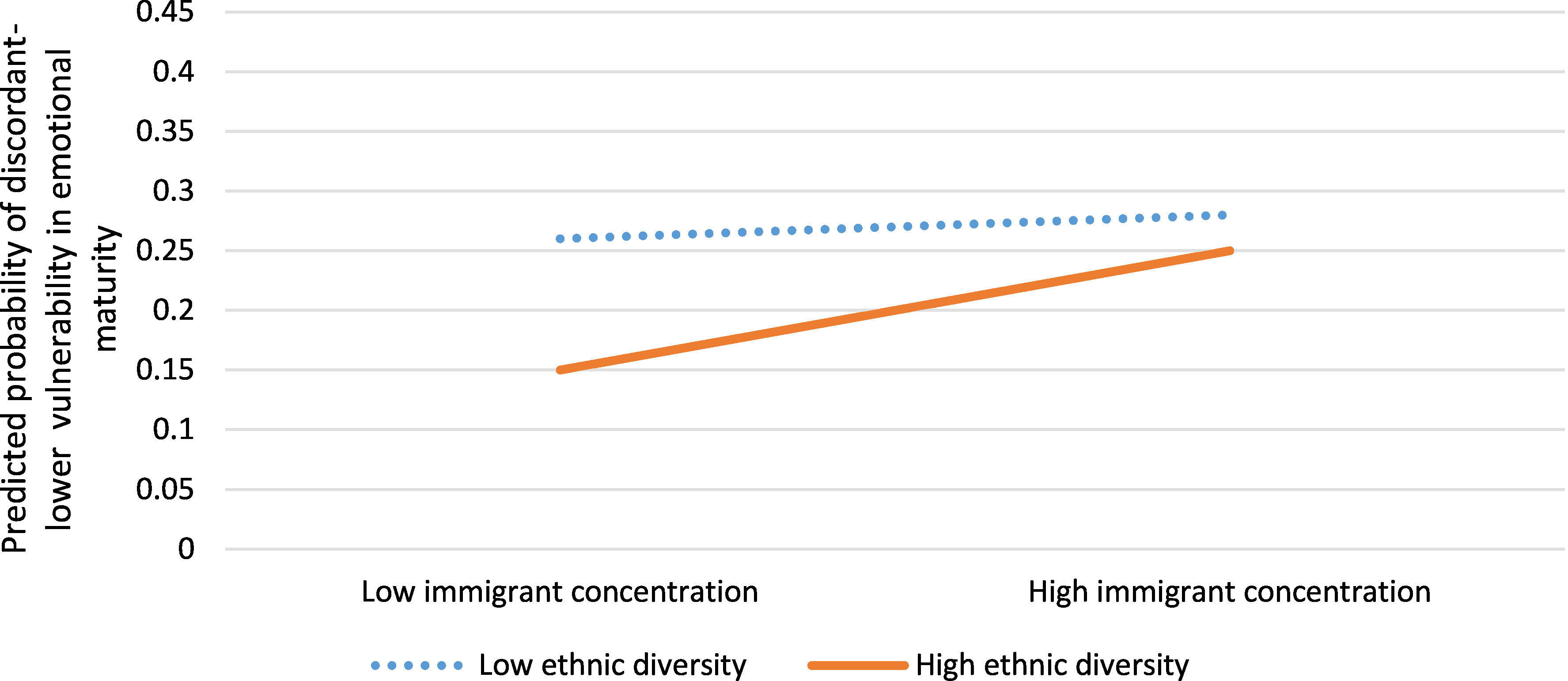


**Figure 5: Interaction between immigrant concentration and ethnic diversity for neighbourhoods with discordant-lower vulnerability in language and cognitive development versus concordant neighbourhoods (n=1,619):**
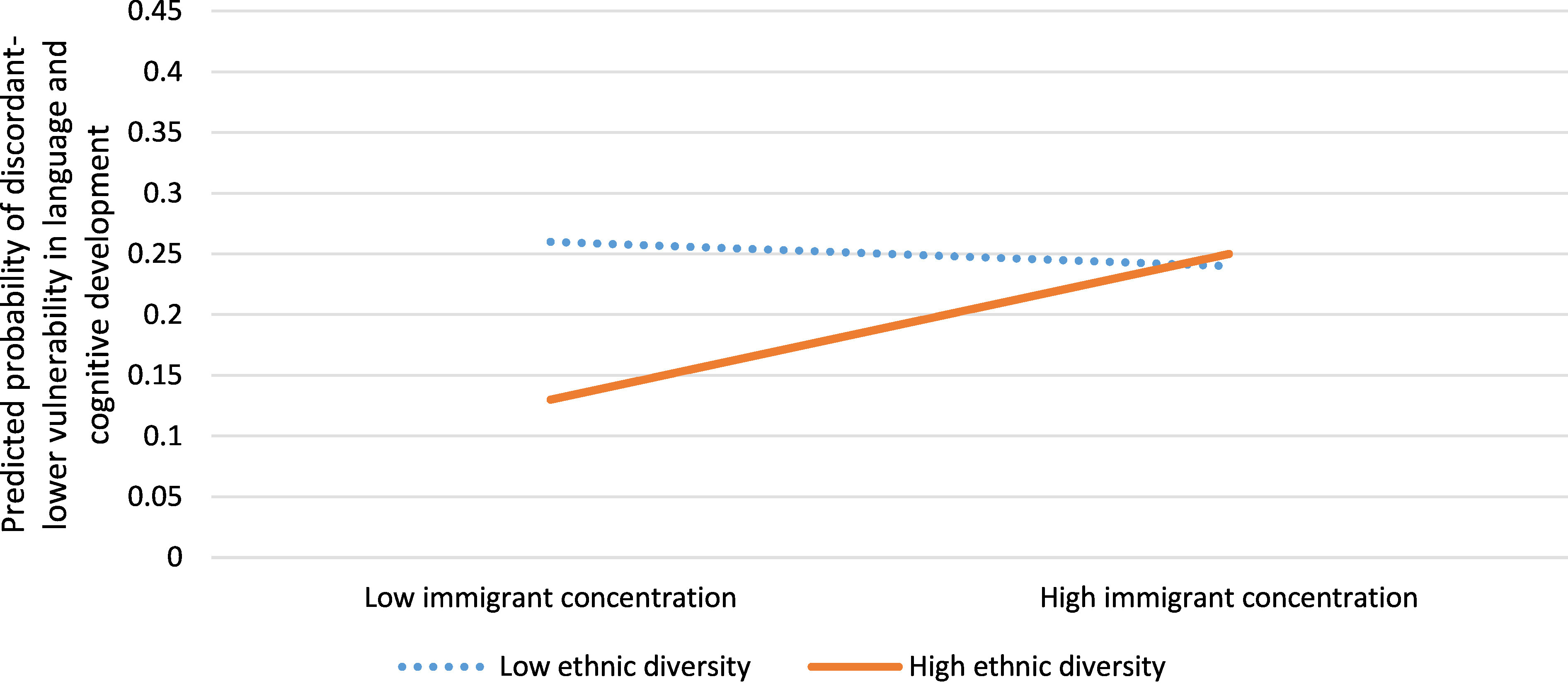


**Figure 6: Interaction between immigrant concentration and ethnic diversity for neighbourhoods with discordant-lower vulnerability in communication skills and general knowledge versus concordant neighbourhoods (n=1,619):**
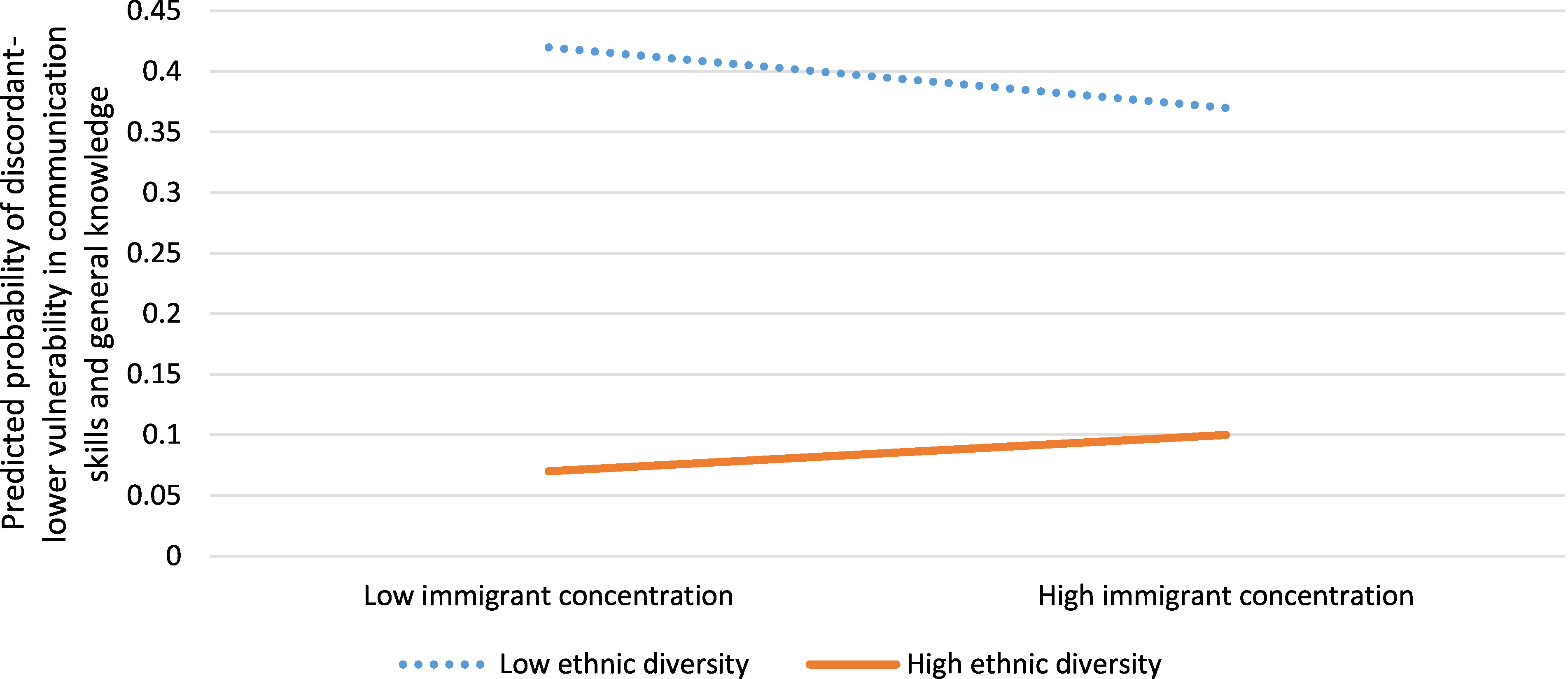


## Abbreviations

aOR	adjusted Odds Ratio
CanNECD	Canadian Neighbourhoods and Early Child Development
CI	Confidence interval
EDI	Early Development Instrument
SES	socioeconomic status
UK	United Kingdom

## References

[1] Forer B, Minh A, Enns JE, Webb S, Duku E, Brownell M, et al A Canadian neighbourhood index for socioeconomic status associated with early child development. Child Ind Res. . 10.1007/s12187-019-09666-y

[2] Carpiano RM, Lloyd JE, C. H. Concentrated affluence, concentrated disadvantage, and children's readiness for school: a population-based, multi-level investigation. Soc Sci Med. 2009;69(3):420-432. . 10.1016/j.socscimed.2009.05.028 19540643

[3] Kershaw P, Irwin L, Trafford K, Hertzman C. The British Columbia atlas of child development. 1st ed (Vol. 40). Vancouver: Human Early Learning Partnership, Western Geographical Press. Vancouver, B.C.: Human Early Learning Partnership, Western Geographical Press; 2005 Available from: http://earlylearning.ubc.ca/media/publications/bcatlasofchilddevelopment_cd_22-01-06.pdf.

[4] Kohen DE, Brooks-Gunn J, Leventhal T, Hertzman C. Neighbourhood income and physical and social disorder in Canada: associations with young children's competencies. Child Dev. 2002;73(6):1844-1860. . 10.1111/1467-8624.t01-1-00510 12487498

[5] Georgiades K, Boyle MH, Duku E. Contextual influences on children's mental health and school performance: the moderating effects of family immigrant status. Child Dev. 2007;78(5):1572-1591. . 10.1111/j.1467-8624.2007.01084.x 17883449

[6] Lloyd JEV, Hertzman C. How neighborhoods matter for rural and urban children's language and cognitive development at kindergarten and Grade 4. J Community Psychol. 2010;38(3):293-313. . 10.1002/jcop.20365

[7] Putnam RD. E pluribus unum: diversity and community in the twenty-first century the 2006 Johan Skytte prize lecture. Scand Political Stud. 2007;30(2):137-174. . 10.1111/j.1467-9477.2007.00176.x

[8] Alesina A, La Ferrara E. Participation in heterogeneous communities. Q J Econ. 2000;115(3):847-904. 10.1162/003355300554935

[9] Saggar S, Somerville W, Ford R, Sobolewska M. The impacts of migration on social cohesion and integration. A final report to the Migration Advisory Committee. London, UK: Government of UK, British Home Office; 2012 Available from: https://pdfs.semanticscholar.org/fee4/8ee5dd6fcf1105c7a2d904beb071dc3013dd.pdf.

[10] Abada T, Hou F, Ram B. Ethnic differences in educational attainment among the children of Canadian immigrants. Can J Soc. 2009;34(1):1-28. . 10.29173/cjs1651

[11] Kawachi I, Berkman LF. Social cohesion, social capital, and health In: Berkman LF, Kawachi I, editors. Social Epidemiology. Toronto: Oxford University Press; 2000.

[12] Carpiano RM. Toward a neighborhood resource-based theory of social capital for health: Can Bourdieu and sociology help? Soc Sci Med. 2006;62(1):165-175. . 10.1016/j.socscimed.2005.05.020 15992978

[13] Minh A, Muhajarine N, Janus M, Brownell M, Guhn M. A review of neighborhood effects and early child development: How, where, and for whom, do neighborhoods matter? Health Place. 2017;46:155-174. . 10.1016/j.healthplace.2017.04.012 28528276

[14] Brown ED, Ackerman BP. Contextual risk, maternal negative emotionality, and the negative emotion dysregulation of preschool children from economically disadvantaged families. Early Educ Develop. 2011;22(6):931-944. . 10.1080/10409289.2010.50803

[15] Callahan KL, Scaramella LV, Laird RD, Sohr-Preston SL. Neighborhood disadvantage as a moderator of the association between harsh parenting and toddler-aged children's internalizing and externalizing problems. J Fam Psychol. 2011;25(1):68-76. . 10.1037/a0022448 21355648 PMC3071255

[16] Singh GK, Ghandour RM. Impact of neighborhood social conditions and household socioeconomic status on behavioral problems among US children. Matern Child Health J. 2012; 16(S158-S169) . 10.1007/s10995-012-1005-z 22481571

[17] Froiland JM, Powell DR, Diamond KE. Relations among neighborhood social networks, home literacy environments, and children’s expressive vocabulary in suburban at-risk families. Sch Psychol Int. 2014;35(4):429-444. . 10.1177/0143034313500415

[18] Riina EM, Martin A, Brooks-Gunn J. Parent-to-child physical aggression, neighborhood cohesion, and development of children's internalizing and externalizing. J Appl Dev Psychol. 2014;35(6):468-477. . 10.1016/j.appdev.2014.04.005 PMC12382824 40881355

[19] Bubier JL, Drabick DA, T. B. Autonomic functioning moderates the relations between contextual factors and externalizing behaviors among inner-city children. J Fam Psychol. 2009;23(4):500-510. . 10.1037/a0015555 19685985 PMC2729513

[20] Moore TG, McDonald M, Carlon L, O'Rourke K. Early childhood development and the social determinants of health inequities. Health Promot Int. 2015;30(Suppl. 2):ii102-ii15. . 10.1093/heapro/dav031 26420806

[21] Leventhal T, Brooks-Gunn J. The neighborhoods they live in: the effects of neighborhood residence on child and adolescent outcomes. Psychol Bull. 2000;126(2):309-337. . 10.1037/0033-2909.126.2.309 10748645

[22] Tucker-Seeley RD, Subramanian SV, Li Y, Sorensen G. Neighborhood safety, socioeconomic status, and physical activity in older adults. Am J Prev Med. 2009;37(3):207-213. . 10.1016/j.amepre.2009.06.005 19595554 PMC3685411

[23] Coen SE, Ross NA. Exploring the material basis for health: characteristics of parks in Montreal neighborhoods with contrasting health outcomes. Health Place. 2006;12:361-371. . 10.1016/j.healthplace.2005.02.001 16814195

[24] Turcotte M. Civic engagement and political participation in Canada. Ottawa, ON: Statistics Canada; 2015 Available from: http://www.statcan.gc.ca/pub/89-652-x/89-652-x2015006-eng.htm.

[25] Guhn M, Janus M, Enns J, Brownell M, Forer B, Duku E, et al Examining the social determinants of children's developmental health: protocol for building a pan-Canadian population-based monitoring system for early childhood development. BJM Open. 2016;6(4) . 10.1136/bmjopen-2016-012020 PMC4853992 27130168

[26] Janus M, Enns J, Forer B, Raos R, Gaskin A, Webb S, et al A pan-Canadian data resource for monitoring child developmental health: The Canadian neighbourhoods early child development (CanNECD) database. IJPDS. 2018;3(3):1-11. . 10.23889/ijpds.v3i3.431 PMC7479929 32935014

[27] Janus M, Brinkman S, Duku E, Hertzman C, Santos R, Sayers M, et al The Early Development Instrument: a population-based measure for communities. A handbook on development, properties, and use. Hamilton, ON: Offord Centre for Child Studies; 2007 Available from: http://www.elcmiamidademonroe.net/Knowledge%20Center/reports/2007_12_FINAL.EDI.HANDBOOK.pdf.

[28] Janus M, Offord D. Development and psychometric properties of the Early Development Instrument (EDI): A measure of children’s school readiness. Can J Behav Sci. 2007;39(1):1-22. . 10.1037/cjbs2007001

[29] Guhn M, Zumbo BD, Janus M, Hertzman C. Special issue: Validation theory and research for a population-level measure of children’s development, wellbeing, and school readiness. Soc Indic Res. 2011;103(2):183-191. 10.1007/s11205-011-9841-6

[30] Thomson KC, Richardson CG, Gadermann AM, Emerson SD, Shoveller J, Guhn M. Association of childhood social-emotional functioning profiles at school entry with early-onset mental health conditions. JAMA Network Open. 2019;2:1-15. . 10.1001/jamanetworkopen.2018.6694 PMC6324314 30646194

[31] Guhn M, Milbrath C, Hertzman C. Associations between child home language, gender, bilingualism and school readiness: A population-based study. Early Child Res Q. 2016;35:95-110. . 10.1016/j.ecresq.2015.11.003

[32] Guhn M, Gadermann A, Zumbo BD. Does the EDI measure school readiness in the same way across different groups of children? Early Educ Develop. 2007;18(3):453-472. . 10.1080/10409280701610838

[33] Kershaw P, Forer B, Lloyd JEV, Hertzman C, Boyce WT, Zumbo BD, et al The use of population‐level data to advance interdisciplinary methodology: a cell‐through‐society sampling framework for child development research. Int J Soc Res Methodol. 2009;12(5):387-403. . 10.1080/13645570802550257

[34] Kohen DE, Leventhal T, Dahinten VS, McIntosh CN. Neighborhood disadvantage: pathways of effects for young children. Child Dev. 2008;79(1):156-169. . 10.1111/j.1467-8624.2007.01117.x 18269515

[35] Browning CR, Burrington LA, Leventhal T, Brooks-Gunn J. Neighborhood structural inequality, collective efficacy, and sexual risk behavior among urban youth. J Health Soc Behav. 2008;49(3):269-285. . 10.1177/002214650804900303 18771063 PMC3111971

[36] Olfert MR, Partridge M. Creating the cultural community: ethnic diversity vs. agglomeration. Spatial Economic Analysis. 2011;6(1):25-55. . 10.1080/17421772.2010.540032

[37] Ottaviano GIP, Peri G. The economic value of cultural diversity: evidence from US cities. J Econ Geogr. 2006;6(1):9-44.

[38] Schaeffer M. Can competing diversity indices inform us about why ethnic diversity erodes social cohesion? A test of five diversity indices in Germany. Soc Sci Res. 2013;42(3):755-774. . 10.1016/j.ssresearch.2012.12.018 23521993

[39] Statistics Canada. Countries and areas of interest for social statistics - SCCAI 2009 Ottawa, ON: Statistics Canada; 2016. Available from: http://www23.statcan.gc.ca/imdb/p3VD.pl?Function=getVD&TVD=127806.

[40] Rothman KJ. No adjustments are needed for multiple comparisons. Epidemiology. 1990;1(1):43-6. 2081237

[41] IBM Corp. IBM SPSS Statistics for Windows, Version 24.0 Armonk, NY: IBM Corp.; 2016.

[42] The Migration Observatory. Immigrantion, diversity and social cohesion. Oxford, UK: University of Oxford; 2017 Available from: https://migrationobservatory.ox.ac.uk/resources/briefings/immigration-diversity-and-social-cohesion/.

[43] Walks RA, Bourne LS. Ghettos in Canada’s cities? Racial segregation, ethnic enclaves and poverty concentration in Canadian urban areas. Can Geogr. 2006;50(3):273-297. . 10.1111/j.1541-0064.2006.00142.x

[44] Kershaw P, Forer B. Selection of area-level variables from administrative data: An intersectional approach to the study of place and child development. Health Place. 2010;16:500-511. . 10.1016/j.healthplace.2009.12.008 20089438

[45] Vaden-Kiernan M, D’Elio MA, O’Brien R, Tarullo LB, Zill N, Hubbell-McKey R. Neighborhoods as a developmental context: a multilevel analysis of neighborhood effects on Head Start families and children. Am J Community Psychol. 2010;45(1-2):49-67. . 10.1007/s10464-009-9279-z 20066488

[46] Jeon L, Buettner CK, Hur E. Family and neighborhood disadvantage, home environment, and children’s school readiness. J Fam Psychol. 2014;28(5):718-727. . 10.1037/fam0000022 25150370

[47] van Hooijdonk C, Droomers M, van Loon JA, van der Lucht F, Kunst AE. Exceptions to the rule: healthy deprived areas and unhealthy wealthy areas. Soc Sci Med. 2007;64(6):1326-1342. . 10.1016/j.socscimed.2006.10.041 17187909

[48] Mitchell R, Gibbs J, Tunstall H, Platt SDa, Dorling D. Factors which nurture geographical resilience in Britain: a mixed methods study. J Epidemiol Community Health. 2009;63:18-23. . 10.1136/jech.2007.072058 18628268

[49] Dominguez S, Watkins C. Creating networks for survival and mobility: social capital among African American and Latin-American low-income mothers. Soc Probl. 2003;50(1):111-135. . 10.1525/sp.2003.50.1.111

[50] Menezes NM, Georgiades K, Boyle MH. The influence of immigrant status and concentration on psychiatric disorder in Canada: a multi-level analysis. Psychol Med. 2011;41(10):2221-2231. . 10.1017/S0033291711000213 21349240

[51] Chen X, Tse HC. Social and psychological adjustment of Chinese Canadian children. Int J Behav Dev. 2010;34(4):330-338. . 10.1177/0165025409337546

[52] Berry JW, Phinney JS, Sam DL, Vedder P. Immigrant youth: acculturation, identity, and adaptation. Appl Psychol. 2006;55(3):303-332. . 10.1111/j.1464-0597.2006.00256.x

[53] Li J. Parental expectations of Chinese immigrants: a folk theory about children's school achievement. Race Ethn Educ. 2004;7(2):166-183. . 10.1080/1361332042000234286

[54] Sue S, Okazaki S. Asian-American educational achievements: a phenomenon in search of an explanation. Am J of Psych. 2009;S(1):45-55. https://migrationobservatory.ox.ac.uk/resources/briefings/immigration-diversity-and-social-cohesion/10.1037//0003-066x.45.8.913 2221563

[55] Fuligni AJ, Fuligni AS. Immigrant families and the educational development of their children In: Lansford JE, Deater-Deckard K, Bornstein MH, editors. Immigrant families in contemporary society. New York: Guilford Press; 2007.

[56] Lu PH, del Canto S, Muhajarine N, Kitchen P, Newbold B, Randall J, et al Quality of life of immigrants: integration experiences among Asian immigrants in Saskatoon. Engaged Scholar Journal: Community-Engaged Research, Teaching, and Learning. 2015;1(2):131-148.

[57] Galster GC. The mechanism(s) of neighbourhood effects: theory, evidence and policy implications In: Van Ham M, Manley D, Bailey N, Simpson L, Maclennan D, editors. Berlin: Springer; 2012.

[58] Telzer EH, Fuligni AJ. Daily family assistance and the psychological well-being of adolescents from Latin American, Asian, and European backgrounds. Dev Psychol. 2009;45(4):1177-1189. . 10.1037/a0014728 19586187

[59] Statistics Canada. Immigration and ethnocultural diversity: key results from the 2016 Census. Ottawa: Statistics Canada; 2017 Available from: www150.statcan.gc.ca/n1/daily-quotidien/171025/dq171025b-eng.htm.

[60] Kennedy S, Kidd MP, McDonald JT, Biddle N. The healthy immigrant effect: patterns and evidence from four countries. JIMI. ;():-2015;16(2):317-332. . 10.1007/s12134-014-0340-x

[61] Hoff E. Bilingual development in children of immigrant families. Child Dev Perspect. 2017;12(2):80-86. . 10.1111/cdep.12262 29805472 PMC5966288

[62] Paradis J. Grammatical morphology in children learning English as a second language: implications of similarities with specific language impairment. Lang Speech Hear Serv Sch. 2005;36:172-187. . 10.1044/0161-1461(2005/019 16175882

[63] Gathercole VCM, Thomas EM. Bilingual first-language development: Dominant language takeover, threatened minority language take-up. Biling Lang Cogn. 2009;12:213-237. . 10.1017/s1366728909004015

[64] May EM, Azar ST, Matthews SA. How does the neighborhood “come through the door?” Concentrated disadvantage, residential instability, and the home environment for preschoolers. Am J Community Psychol. 2018; 61(1-2). . 10.1002/ajcp PMC5837934 29315625

[65] Pearson AL, Pearce J, Kingham S. Deprived yet healthy: neighbourhood-level resilience in New Zealand. Soc Sci Med. 2013;91:238-245. . 10.1016/j.socscimed.2012.09.046 23232023

